# First pediatric experience of SL-401, a CD123-targeted therapy, in patients with blastic plasmacytoid dendritic cell neoplasm: report of three cases

**DOI:** 10.1186/s13045-018-0604-6

**Published:** 2018-05-02

**Authors:** Weili Sun, Huaying Liu, Young Kim, Nicole Karras, Anna Pawlowska, Debbie Toomey, Wade Kyono, Paul Gaynon, Joseph Rosenthal, Anthony Stein

**Affiliations:** 1 Department of Pediatrics, City of Hope National Medical Center, 1500 E. Duarte Road, Duarte, CA 91010 USA; 2 Department of Pediatrics, Nanfang Hospital, Southern Medical University, Guangzhou, China; 3 Department of Pathology, City of Hope National Medical Center, Duarte, CA USA; 4 Department of Pediatrics, Kapiolani Medical Center for Women and Children, University of Hawaii, Honolulu, HI USA; 5 Children’s Center for Cancer and Blood Diseases, Children’s Hospital Los Angeles, University of Southern California, Los Angeles, CA USA; 6 Department of Hematology and Hematopoietic Cell Transplantation, City of Hope National Medical Center, Duarte, CA USA

**Keywords:** Pediatric, BPDCN, SL-401, Targeted therapy

## Abstract

**Background:**

Blastic plasmacytoid dendritic cell neoplasm (BPDCN) is a highly aggressive hematological malignancy with extremely poor outcome. The median overall survival for adult patients is 9–13 months. Pediatric patients are exceedingly rare with an unclear clinical course. Currently, no standardized therapy has been established, although an acute lymphoblastic leukemia type of treatment appears to be more effective in those patients who are able to tolerate aggressive chemotherapy. SL-401 is a targeted therapy directed to CD123, a protein ubiquitously expressed at high level on the surface of BPDCN blasts. In adult phase 2 trials, it has demonstrated efficacy with 90% overall response rate. No pediatric patients with BPDCN using SL-401 have been reported.

**Case presentation:**

Here, we report the first pediatric experience of three children with BPDCN treated with SL-401 at our institution. All patients tolerated SL-401 without significant toxicities. One patient with multiply relapsed and refractory disease had no response. The other two cases had significant and rapid clinical improvement after the two courses of treatment. However, the response was transient, and growth of soft tissue mass was observed in-between cycles in both patients with large tumor burden.

**Conclusions:**

This is the first report of SL-401 in pediatric patients with BPDCN. Sl-401 was well tolerated and can produce a promising response. Further testing this agent in children is warranted.

## Background

Blastic plasmacytoid dendritic cell neoplasm (BPDCN) is a highly aggressive hematological malignancy that primarily involves bone marrow (BM) and/or skin; secondary sites include lymph nodes, soft tissue, and central nervous system [1, 2]. BPDCN is derived from the precursors of plasmacytoid dendritic cells and characterized by the expression of CD123, CD4, and CD56, and the absence of other lineage markers [1, 2]. BPDCN typically affects elderly patients; however, it can be present at any age [3]. Pediatric patients are exceedingly rare with an unclear clinical course [3, 4]. Currently, no standardized therapeutic approach has been established, although an acute lymphoblastic leukemia (ALL) type of treatment appears to be more effective in those patients who are able to tolerate aggressive chemotherapy [2–4]. The prognosis of BPDCN is extremely poor. Most adult patients relapse in less than 2 years, and the median overall survival is 9–13 months [2, 5]. Allogeneic stem cell transplant (SCT) in the first remission could produce durable remission in adult patients [6]. The role of SCT in children is not clear [3, 7, 8].

CD123, interleukin 3 (IL-3) receptor α chain, is ubiquitously expressed at high level on the surface of BPDCN blasts, making it an attractive therapeutic target [1]. As a targeted therapy directed to CD123, SL-401 is a recombinant fusion protein that links IL-3 to a truncated diphtheria toxin (DT) payload (IL-3 replaces the native binding domain of DT) [9]. After binding to CD123, SL-401 becomes internalized, the catalytic domain of DT translocates to the cytoplasm, leading to inhibition of protein synthesis and cell death [9]. In the largest adult trial in BPDCN, SL-401 was generally effective and well tolerated as reported at American Society of Hematology Annual Meeting 2017 [10]. The most common treatment-related adverse events (AEs) were transient transaminitis (50–52%), hypoalbuminemia (50%), thrombocytopenia (38%), fever (29%), and chills (29%). Capillary leak syndrome (CLS) occurred in 19% of patients [9]. At the optimal dose of 12 μg/kg/day, there were 2/119 (1.7%) grade 5 events of CLS across all SL-401 trials (BPDCN, acute leukemia, myeloproliferative neoplasms, and multiple myeloma) [9]. In the ongoing BPDCN phase 2 trial, the overall response rate (ORR) at a dose of 12 μg/kg/day was 90% (26/29) with a 72% rate of CR + CRc + CRi (CR = complete response; CRc = clinical CR [CR with minimal residual skin abnormality]; CRi = CR with incomplete hematologic recovery) in newly diagnosed patients. In patients with relapsed and/or refractory (R/R) disease, the ORR was 69% (9/13) with a 38% rate of CR + CRc + CRi. In addition, 45% (13/29) of patients in the first remission were subsequently bridged to SCT after receiving SL-401 [11].

Up to date, no pediatric patients with BPDCN using SL-401 have been reported. Here, we report the first experience of three children with BPDCN treated with SL-401 at City of Hope (COH) (Table 1). All patients received a 5-day infusion of 12 μg/kg/day SL-401 every 2–3 weeks. Each patient received single patient IND approval. All signed an informed consent/assent. This retrospective study has been approved by an institutional review board of COH.

**Table 1:** Clinical presentation, immunophenotype, and cytogenetics

Age/gender	Initial sites of involvement	Immunophenotype						Cytogenetics	Molecular study
		CD4	CD56	CD123	TdT	MPO	Other positive		
10 years/F	Skin, BM	+	+	+	–	–	CD43, CD45, TCL-1, S100	45, XX, *t* (1;6), (q21; q23), − 9, add (11) (q23), + 16, − 21	N/A
12 years/F	Skin, LN	+	+	+	Focal	–	CD43, CD45, TCL-1, S100, CD99	46, XX	NRAS G12R, Myb-PLEKH01
15years/F	SC, BM	+	+	+	+	–	CD45, CD5,CD7, CD11c, CD38, CD64	46, XX, *t* (12;18) (p13; q21), chr. 6 tetraploidy, ETV6 del	N/A

## Case presentation

### Case 1

A 10-year-old girl presented with a soft tissue mass in the right anterior thigh and BM involvement. She was treated with high risk ALL therapy. The patient suffered first combined (BM and central nervous system [CNS]) relapse at 22 months and achieved a second CR after first salvage therapy. Due to invasive fungal infection, she subsequently received 4 cycles of decitabine and was found to have second BM and CNS relapse 5 months later. She received second salvage therapy with fludarabine and cytarabine, then transferred care to COH. Upon arrival, her BM was in CR, and minimal residual disease (MRD) was positive at 0.37%, with a strong signal of CD123. CSF was negative. However, a new small scalp mass was noted.

She received SL-401 treatment as a bridging therapy while an unrelated donor search was ongoing. During course 1, she developed facial flushing and fever that resulted in a 24-h delay of the third dose. At the end of course 1, she had stable scalp lesions and 0.33% BM MRD. During course 2, she developed tachycardia, hypoxia, and urgency to evacuate bowl shortly after the second dose. The symptoms improved quickly after oxygen and diphenhydramine. She tolerated subsequent doses with premedication. However, the scalp lesion enlarged and was confirmed positive for BPDCN. End of course 2 BM showed 80% blast. Blasts from both scalp lesion and BM remained to be CD123 positive.

SL-401 was discontinued. She did not respond to two additional salvage chemotherapies with SMILE (dexamethasone, methotrexate, ifosfamide, asparaginase, and etoposide) and CEC (clofarabine, etoposide, and cyclophosphamide). The patient died 5 months later from progressive disease.

### Case 2

**Fig. 1:**
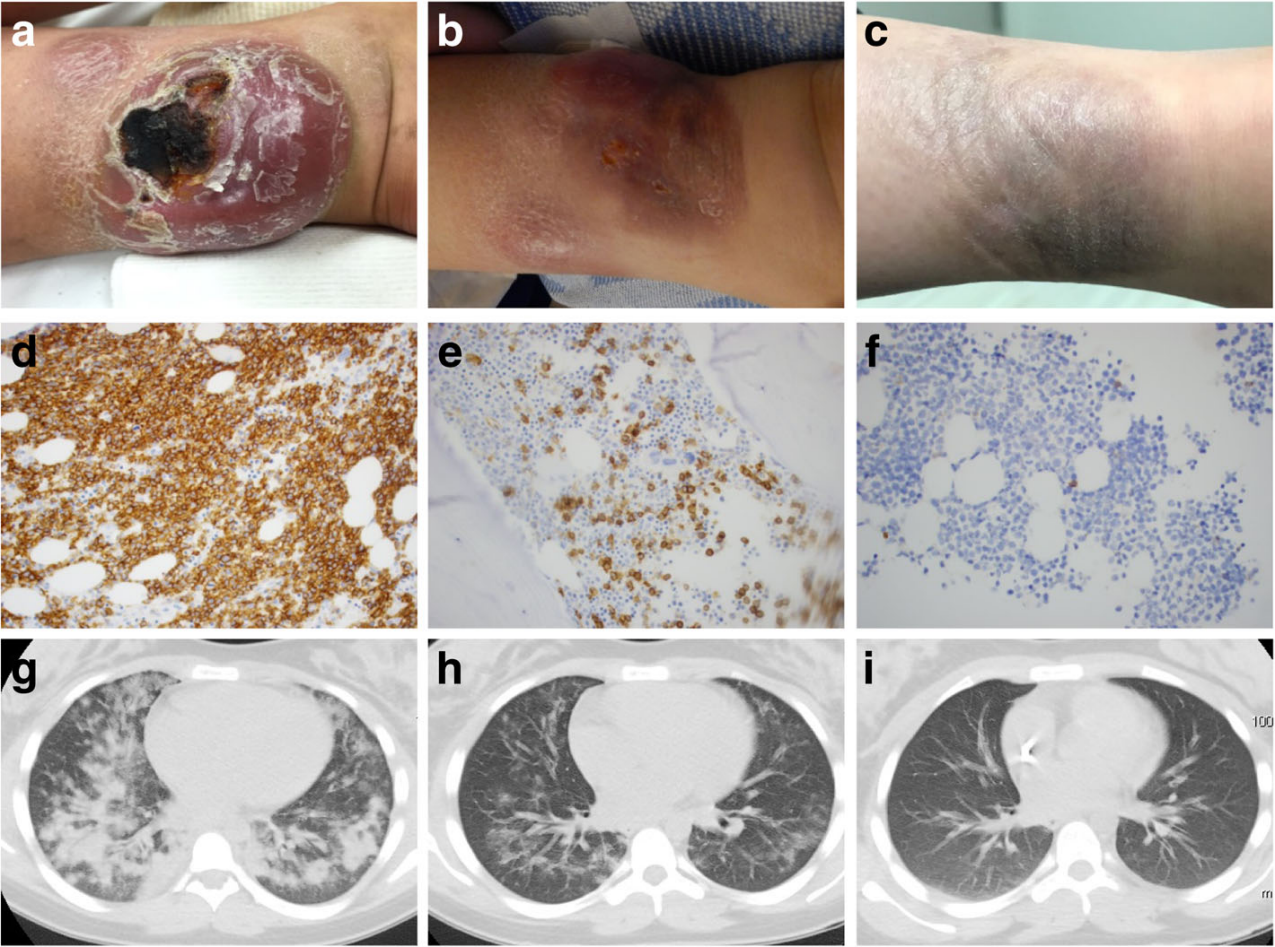
Patient response. Left ankle mass of patient #2 pre-treament (**a**), post two courses of SL-401 (**b**), and post-HCT (**c**). Bone marrow CD123 staining and chest CT of patient #3 pre-treatment (**d**, **g**), post one course of SL-401 (**e**, **h**), and post two courses of SL-401 (**f**, **i**)

A 12-year-old girl presented with localized, progressive soft tissue mass of the posterior medial left ankle and no BM involvement. The family initially refused chemotherapy but agreed to SL-401. She received five courses of SL-401 without any AEs. She was in school full time during the treatment. There was a visual decrease in the size of the mass. At the end of course 2, the mass had decreased from the size of a large grapefruit to approximately 2 × 3 cm (Fig. 1a, b). However, during cycles 4 and 5, the mass was noted to grow between cycles. After discussion, the family agreed to chemotherapy. CR was achieved after 2 cycles of hyper CVAD. She received 1800 cGy radiation to the mass, followed by SCT from an unrelated, partially HLA-matched cord blood unit. The conditioning regimen included total body irradiation, cyclophosphamide, and fludarabine. She tolerated SCT without significant toxicities. Currently, she is 12 months post-SCT, off all immune suppression, remains in remission, and is doing well.

### Case 3

A 15-year-old girl presented with periorbital ecchymoses and BM involvement. She was treated per high risk ALL therapy and achieved MRD negative CR. She was noted to be non-compliant to the treatment. She experienced first BM relapse at 12 months and was refractory to the first salvage therapy with BDMV (bortezomib, dexamethasone, mitoxantrone, and vinorelbine) [12]. She started the second salvage therapy with CEC, but only received 80% of planned chemotherapy due to patient refusal. The peripheral blast disappeared with full myelorecovery. Three months later, the patient had a reappearance of the peripheral blast and agreed to SL-401 treatment at COH.

Pretreatment disease evaluation revealed 43% circulating blasts, chest and abdominal lymphadenopathy, and extensive lung involvement (Fig. 1d, g). During course 1, the patient experienced pyrexia, hypoxia, and shortness of breath, which resolved after diphenhydramine. The fifth dose was held due to hypoalbuminemia. At the end of course 1, she had a partial response with the disappearance of the peripheral blast, a reduction of BM blast from 90 to 20%, and marked improvement of the pulmonary disease (Fig. 1e, h). Course 2 was delayed for 3 weeks because the patient did not return for treatment. She was noted to have a new skin rash on the chest wall and cervical and axillary lymphadenopathy that was confirmed to be BPDCN, CD123 positive prior to course 2. During course 2, she again experienced transient fever and mild peripheral edema. The fifth dose was held due to a 5-kg weight gain. There was a marked improvement of the skin rash and lymphadenopathy after course 2. The BM showed morphological remission, and she had complete resolution of pulmonary lesions (Fig. 1f, i). However, new lesions were noted at her breast and orbital area. Therefore, cycle 3 was given a few days early, and she tolerated it without toxicity. The extramedullary disease remained stable. Cycle 4 started on day 14 and was well tolerated. Unfortunately, the extramedullary disease progressed.

SL-401 was discontinued, and the patient planned to receive total marrow and lymphoid irradiation followed by haploidentical SCT [13]. After 2 days of conditioning the regimen, the patient and her family decided to withdraw care. She died from a progressive disease a few months later.

## Discussion and conclusions

BPDCN is a highly aggressive hematological malignancy. In the past, despite intensive combination chemotherapy, most adult patients die within 2 years of diagnosis [2, 5]. The published reports in children are extremely limited. One small case series reported 61% progression-free survival [3]. No consensus on the treatment options has been established.

SL-401, a targeted therapy directed to CD123, has demonstrated a tolerable safety profile and robust activity in adult patients with BPDCN. Here, we report the first pediatric experience with SL-401. Similar to adults, we observed mild, transient infusion-related AEs that were easily manageable. One patient with large tumor burden development hypoalbuminemia and weight gain, suggesting possible development of CLS. Despite that, this patient tolerated more frequent dosing. Another patient was able to maintain an excellent quality of life and attended school full time. Overall, the therapy is very well tolerated.

Two of the three patients responded to SL-401. None of them achieved CR. Unlike in adults where single-agent SL-401 activity is quite robust and durable; the response was transient, and the growth of soft tissue mass was observed in-between cycles in both responding patients who had large tumor burden. It is not clear why the response was less durable in our patients when compared to what was observed in adults. We speculate that there might be underlying biological differences between children and adults, and additional studies with more patients are needed. Our experience suggests that in children, SL-401 might be considered for combination therapy; albeit this study represents very few patients to draw definitive conclusions on single agent versus combination.

Of note, tumors from patient #1 and #3 remained to be CD123 positive despite clinical evidence of disease progression. This was consistent with what was observed in the adult study, suggesting that the mechanism of resistance was not due to the loss or decreased expression of CD123 [14] Recently, Stephansky et al. demonstrated that azacitidine could reverse the acquired resistance and synergize with SL-401 in the pre-clinical study [14]. This hypothesis is currently tested in an investigator-initiated trial [14]. Additionally, it has been reported that BPDCN blast is dependent on BCL2 and is sensitive to venetoclax [15]. It may be beneficial to evaluate whether the combination of SL-401 with azacitidine, BCL2 inhibitors, or other chemotherapy or using a more intensive treatment schedule such as every 2 weeks is tolerated and generate a more durable response in children. In addition, administering SL-401 to patients with low disease burden, as a bridging therapy prior to SCT or as maintenance therapy, needs to be further investigated.

In summary, this is the first report of SL-401 in pediatric patients with BPDCN. SL-401 was well tolerated and can produce a promising response. Further testing this agent in children is warranted.

## Abbreviations

AE: Adverse event
ALL: Acute lymphoblastic leukemia
BM: Bone marrow
BPDCN: Blastic plasmacytoid dendritic cell neoplasm
CEC: Clofarabine, etoposide, cyclophosphamide
CLS: Capillary leak syndrome
CNS: Central nervous system
COH: City of Hope
CR: Complete response
CRc: Clinical complete response
CRi: Complete response with incomplete hematologic recovery
DT: Diphtheria toxin
MRD: Minimal residual disease
ORR: Overall response rate
R/R: Relapsed and/or refractory
SCT: Stem cell transplant
